# Targeting CD10 on B-Cell Leukemia Using the Universal CAR T-Cell Platform (UniCAR)

**DOI:** 10.3390/ijms23094920

**Published:** 2022-04-28

**Authors:** Nicola Mitwasi, Claudia Arndt, Liliana R. Loureiro, Alexandra Kegler, Frederick Fasslrinner, Nicole Berndt, Ralf Bergmann, Vaclav Hořejší, Claudia Rössig, Michael Bachmann, Anja Feldmann

**Affiliations:** 1 Helmholtz-Zentrum Dresden-Rossendorf, Institute of Radiopharmaceutical Cancer Research, Bautzner Landstraße 400, D-01328 Dresden, Germany; n.mitwasi@hzdr.de (N.M.); c.arndt@hzdr.de (C.A.); l.loureiro@hzdr.de (L.R.L.); a.kegler@hzdr.de (A.K.); n.berndt@hzdr.de (N.B.); r.bergmann@hzdr.de (R.B.); a.feldmann@hzdr.de (A.F.); 2 Mildred Scheel Early Career Center, Faculty of Medicine Carl Gustav Carus, TU Dresden, D-01307 Dresden, Germany; frederick.fasslrinner@uniklinikum-dresden.de; 3 Medical Clinic and Polyclinic I, University Hospital Carl Gustav Carus, TU Dresden, D-01307 Dresden, Germany; 4 Department of Biophysics and Radiation Biology, Semmelweis University, H-1094 Budapest, Hungary; 5 Institute of Molecular Genetics of the Academy of Sciences of the Czech Republic, 142 20 Prague, Czech Republic; vaclav.horejsi@img.cas.cz; 6 Department of Pediatric Hematology and Oncology, University Children’s Hospital Münster, 48149 Münster, Germany; rossig@uni-muenster.de; 7 National Center for Tumor Diseases (NCT), D-01307 Dresden, Germany; 8 German Cancer Consortium (DKTK), Partner Site Dresden and German Cancer Research Center (DKFZ), 69120 Heidelberg, Germany; 9 Tumor Immunology, University Cancer Center (UCC), University Hospital Carl Gustav Carus, TU Dresden, D-01307 Dresden, Germany

**Keywords:** CD10, immunotherapy, CAR T-cells

## Abstract

Chimeric antigen receptor (CAR)-expressing T-cells are without a doubt a breakthrough therapy for hematological malignancies. Despite their success, clinical experience has revealed several challenges, which include relapse after targeting single antigens such as CD19 in the case of B-cell acute lymphoblastic leukemia (B-ALL), and the occurrence of side effects that could be severe in some cases. Therefore, it became clear that improved safety approaches, and targeting multiple antigens, should be considered to further improve CAR T-cell therapy for B-ALL. In this paper, we address both issues by investigating the use of CD10 as a therapeutic target for B-ALL with our switchable UniCAR system. The UniCAR platform is a modular platform that depends on the presence of two elements to function. These include UniCAR T-cells and the target modules (TMs), which cross-link the T-cells to their respective targets on tumor cells. The TMs function as keys that control the switchability of UniCAR T-cells. Here, we demonstrate that UniCAR T-cells, armed with anti-CD10 TM, can efficiently kill B-ALL cell lines, as well as patient-derived B-ALL blasts, thereby highlighting the exciting possibility for using CD10 as an emerging therapeutic target for B-cell malignancies.

## 1. Introduction

Chimeric antigen receptor (CAR)-expressing T-cells have indeed shown a remarkable potential for the treatment of various B-cell related malignancies, which has led to the approval of several CAR T-cell products such as Kymriah^TM^, Yescarta^TM^ and others [1,2,3]. However, clinical experience has shown that targeting single antigens, such as CD19, on leukemic cells can result in relapse with antigen-negative leukemic clones following the treatment [4,5,6]. Therefore, it became crucial to find additional targets, which are widely expressed in B-cell malignancies, in order to allow subsequent or combinational targeting of leukemic cells, and thereby avoid relapse. Another vital aspect that needs to be addressed in regards to CAR T-cell therapy, is the risk of developing severe side effects, such as cytokine release syndrome or neurological toxicities, which could be life threating [7,8,9].

In our paper, we tackled both issues by investigating the feasibility of targeting the common acute lymphoblastic leukemia antigen, also known as CD10, with our switchable UniCAR technology. CD10 is known to be a transmembrane metalloprotease that cleaves and inactivates a variety of functional neuro- and hormonal peptides [10,11]. It is present in common lymphoid progenitors and several stages of B-cell development [12,13,14]. CD10 is considered an interesting therapeutic target, due to its expression on a variety of B-cell malignancies, including B-cell acute lymphoblastic leukemia (B-ALL) [14,15]. In fact, CD10 was first identified as a common ALL antigen, due to the reaction of its anti-serum with the majority of non T-cell ALL cells [16]. Moreover, it was found to be expressed in lymphomas, such as Follicular lymphoma, Burkitt lymphoma and others, which makes it a commonly used marker for the classification and differentiation of these various B-cell malignancies [17,18,19,20,21,22,23]. Interestingly, CD10 expression is not limited to hematological malignancies, and has also been shown to be overexpressed in several solid tumors, such as melanoma, colorectal cancer, prostate cancer, thyroid carcinoma and others [24,25,26,27,28,29,30,31,32,33]. Furthermore, it has been found to be correlated with prognosis, tumor metastasis and aggressiveness [30,31,34,35,36,37]. Based on the aforementioned facts, CD10 represents a valuable tumor target, especially for immune-based therapies, such as CAR T-cells [38,39].

**Figure 1:**
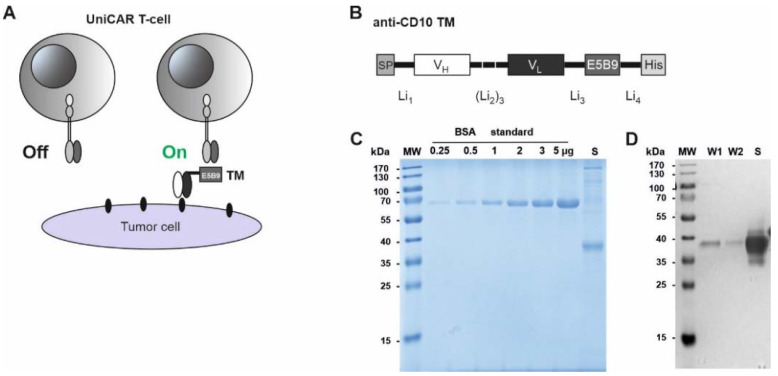
Design and expression of anti-CD10 target module. (**A**) Schematic representation of the UniCAR system; UniCAR T-cells can only be activated in the presence of the target module (TM) and their target cell (On). In the absence of the TM, the UniCAR T-cells are switched off (Off). (**B**) The anti-CD10 TM was obtained by joining the variable light and heavy (V_L_, V_H_) chain domains of CD10 monoclonal antibody (mAb) via peptide linkers followed by fusion of the E5B9 epitope and a hexa-histidine (6xHis) tag. (**C**) The purified TM was analyzed by SDS-PAGE along with bovine serum albumin (BSA) standard, followed by Quick Coomassie^®^ Stain. (**D**) Alternatively, the separated proteins were blotted on nitrocellulose membrane and detected with mouse anti-penta-His Ab and the reaction of the alkaline phosphatase-conjugated to secondary anti-mouse IgG Ab. W1, W2: washes of Ni-NTA columns after purification; S: sample elution (anti-CD10 TM), MW: Molecular weight marker, Li: Linker, SP: Igκ signal peptide.

Since CD10 is also expressed in certain healthy tissues, like the kidneys, epithelial cells of the prostate, liver and others [10,11], targeting it with conventional CAR T-cells needs an improved safety approach, which could be achieved with our switchable UniCAR technology. The UniCAR system is a modular system composed of UniCAR T-cells that cannot recognize surface antigens unless a second soluble adaptor molecule—the target module (TM)—is present [40,41]. In general, TMs consist of a target-binding domain to which a peptide sequence is fused as an epitope tag. Commonly, we use, as a tag, the continuous peptide epitope E5B9, that is part of the primary sequence of the human nuclear protein La-SS/B [42,43]. As the extracellular antigen-binding domain of the UniCAR is derived from the anti-La monoclonal antibody (mAb) 5B9, it recognizes the E5B9 epitope of the respective TM. With its binding site, the TM binds to a tumor antigen. The resulting cross-linkage finally leads to the activation of UniCAR T-cells and, subsequently, to the elimination of tumor cells (Figure 1A). Steering of the UniCAR T-cell activity is based on the concentration and half-life of the TM, which is usually short in order to allow a fast on/off switch and thereby control unwanted side effects (Figure 1A). Previously, we have proven the efficiency of the UniCAR system in targeting a wide range of hematological and solid tumors, both in pre-clinical and clinical studies [44,45,46,47,48,49,50,51,52]. Here, we have designed and created a TM, based on a CD10 mAb, and provided proof of concept for the efficient targeting of a CD10-expressing B-ALL cell line, as well as patient-isolated ALL blasts using the UniCAR system.

## 2. Results

### 2.1. Expression and Purification of Anti-CD10 Target Modules

The sequences of the variable light and heavy domains (V_L_ and V_H_) of an anti-CD10 mAb were determined by sequencing of amplified cDNA that was prepared from hybridoma cells MEM-78. The V_H_ and V_L_ domains were linked via flexible glycine-serine linkers. We fused the E5B9 epitope, followed by a hexa-histidine (6xHis) tag, C-terminally of the resulting single-chain fragment variable (scFv), to allow purification with Ni-NTA affinity chromatography. At the N-terminus, the signal peptide sequence of murine Igκ was added, in order to allow the secretion of the TM (Figure 1B).

Following purification of the TMs from the culture medium of transduced 3T3 cells, the eluted fractions were analyzed on SDS-PAGE and stained with Quick Coomassie^®^ Stain (Figure 1C). Alternatively, the proteins were immunoblotted on a nitrocellulose membrane, and detected with a mouse anti-penta-His Ab (Figure 1D). The full-length TM was detected on the Coomassie-stained gel as well as on the Western blot. The theoretical size of the TM is around 30 kDa. However, it runs slightly higher on the SDS-PAGE gel, as shown in Figure 1C,D, which hints to a post-translational modification of the molecule, or aberrant mobility on the gel. Most importantly, the TM was successfully purified and could be used for further functional analysis.

### 2.2. The Novel Anti-CD10 Target Module Binds Effectively to B-ALL Cell Line

**Figure 2:**
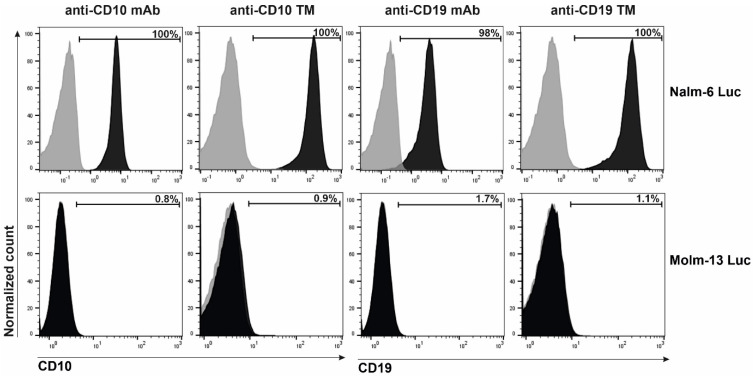
Binding of the novel anti-CD10 TM to Nalm-6 cells. Nalm-6 Luc or Molm-13 Luc cells were stained with either VioBlue-conjugated anti-CD10 mAb, anti-CD10 TM, VioBlue-conjugated anti-CD19 mAb or anti-CD19 TM. The binding of the TMs was then detected using anti-La mAb (clone 5B9) followed by anti-mouse-IgG-Alexa Fluor 647. Binding is represented by a shift in the signal (dark gray). As a negative control, cells were stained with either VioBlue-conjugated isotype control or with anti-La mAb and the detection Ab without the TM (light gray). Binding is indicated as a percentage (%) of positively stained cells.

The ability of anti-CD10 TM to bind to the CD10-expressing B-ALL cell line (Nalm-6) was detected with anti-La mAb (clone 5B9) specific for the E5B9 tag in the TM. As shown in Figure 2, the TM was able to bind to all the Nalm-6 Luc cells, which were confirmed to express CD10 on their surface by staining with an anti-CD10 mAb (Figure 2, upper panel). In addition, we used the previously established anti-CD19 TM as a positive control for the binding [44]. The specificity of the anti-CD10 TM was confirmed on the CD10-negative cell line Molm-13 Luc, where no binding of the TM was detected (Figure 2, lower panel).

### 2.3. UniCAR T-Cells Armed with Anti-CD10 Target Module Exert Specific In Vitro Cytotoxicity against Leukemic Cell Line

**Figure 3:**
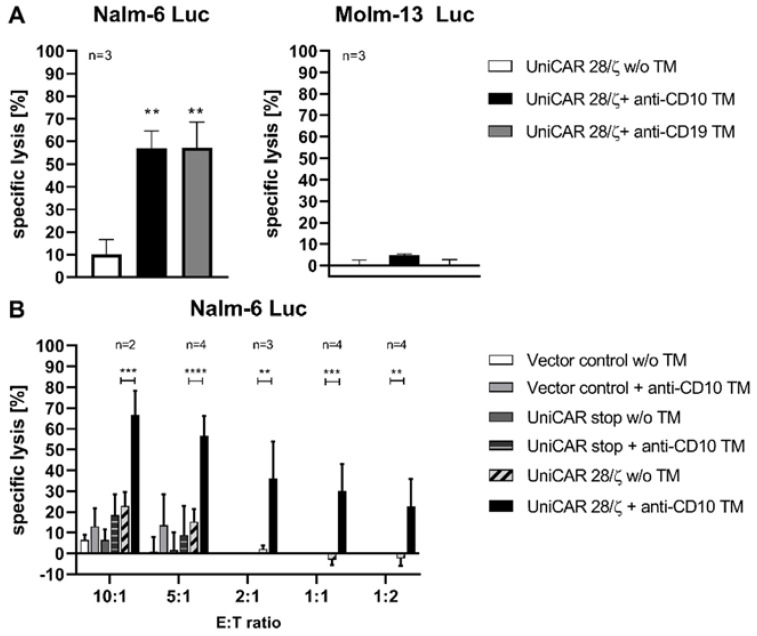
Targeting Nalm-6 Luc cells with UniCAR T-cells armed with anti-CD10 TM. (**A**) Either Nalm-6 Luc or Molm-13 Luc cells were incubated with UniCAR T-cells in the addition of 50 nM anti-CD10 or anti-CD19 TM at 5:1 effector to target cell (E:T) ratio (** p < 0.0021, comparison to sample w/o TM; One-way ANOVA with Dunnett’s multiple comparison test). (**B**) Nalm-6 Luc cells were incubated with different ratios of UniCAR T-cells for 7 h in the absence, or presence, of 50 nM of the anti-CD10 TM. The Vector control (expressing EGFP only), or the UniCAR stop (lacking signaling domains) T-cells were included as controls at the two highest E:T ratios. The cytotoxic effect was then evaluated using a luminescence-based assay. Results are shown as mean ± SD for two to four independent T-cell donors as indicated in the figure (** p < 0.0021, *** p < 0.0002, **** p < 0.0001, comparison to samples w/o TM; Two-way ANOVA with Bonferroni’s multiple comparison test).

In order to investigate the specific cytotoxic activity of armed UniCAR T-cells, they were incubated with the CD10-expressing cell line Nalm-6 Luc or the CD10-negative cell line Molm-13 Luc (Figure 2). As demonstrated in Figure 3A, the UniCAR T-cells (UniCAR 28/ζ) redirected with anti-CD10 TM caused specific and significant lysis of Nalm-6 Luc cells, whereas no significant lysis was observed with Molm-13 Luc cells that lack the targets CD10 and CD19. As a positive control, we included a previously established anti-CD19 TM [44], which resulted in a lysis of the target cells at a level comparable to the anti-CD10 TM.

In addition, the ability of UniCAR T-cells to target the Nalm-6 Luc cell line was evaluated at different effector to target cell ratios (E:T) in the presence, or absence, of the novel TM against CD10. As shown in Figure 3B, redirected UniCAR T-cells effectively, and significantly killed CD10-expressing Nalm-6 Luc cells in the presence of the anti-CD10 TM, even at low E:T ratios. The killing effect further increased with increasing E:T ratios. In contrast, no significant target cell lysis occurred in the absence of the TM. In this experiment, Vector control T-cells (lacking the UniCARs), or non-signaling UniCAR stop T-cells, were used as controls. As observed, these controls caused only a slight killing background when tested for the highest two E:T ratios, regardless of the presence, or absence, of the anti-CD10 TM (Figure 3B). In conclusion, UniCAR T-cells armed with anti-CD10 TM kill CD10-expressing leukemic cells in a target-specific manner.

### 2.4. Estimation of Effective Working Concentrations of Anti-CD10 Target Module

**Figure 4:**
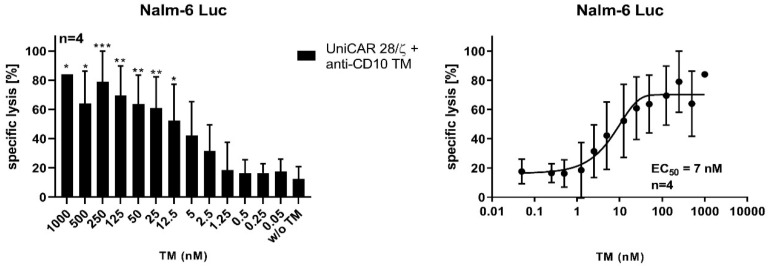
Estimation of effective working concentrations of anti-CD10 TM. In order to determine the half-maximal effective concentration (EC_50_) of the anti-CD10 TM, Nalm-6 Luc cells were incubated with UniCAR T-cells at an E:T ratio of 5:1 in the presence of a range of anti-CD10 TM concentrations (1000 nM to 0.05 nM) for 7 h. The killing was then evaluated using luminescence-based assay. Results are shown as mean ± SD of four independent T-cell donors (* p < 0.0332, ** p < 0.0021, *** p < 0.0002, comparison to sample w/o TM; One-way ANOVA with Dunnett’s multiple comparison test).

UniCAR T-cells kill tumor cells dependent on the concentration of the specific TM, which plays an important role in the efficacy and controllability of the UniCAR system. Here, we have tested the UniCAR T-cells with a range of TM concentrations in order to determine the functional window. A shown in Figure 4, the killing activity of UniCAR T-cells can be titrated according to the TM concentration. A nanomolar range of the TM is needed to activate UniCAR T-cells to kill Nalm-6 Luc cells. The half-maximal effective concentration (EC_50_) was estimated to be around 7 nM.

### 2.5. Redirected UniCAR T-Cells Release Cytokines upon Engaging with CD10-Expressing Leukemia Cells

Secretion of cytokines is another vital function of T-cells, which allows the regulation of other immune components that are involved in the anti-tumor response. Here, we have investigated the ability of UniCAR T-cells to release pro-inflammatory cytokines upon engaging with Nalm-6 Luc cells in the presence of anti-CD10 TM and anti-CD19 TM.

**Figure 5:**
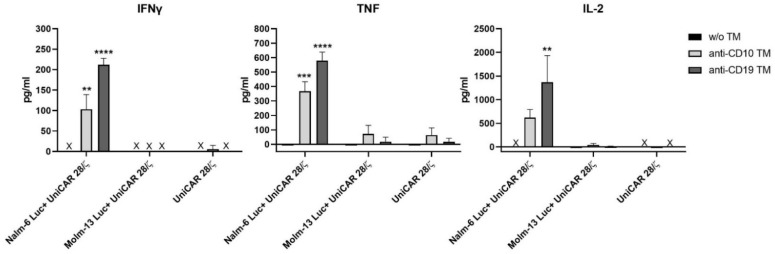
Cytokine release from UniCAR T-cells upon engaging with Nalm-6 Luc cells via anti-CD10 TM. UniCAR T-cells were incubated for 7 h, either alone or in the presence of one of the cell lines (Nalm-6 Luc or Molm-13 Luc), at 5:1 E:T ratio. There was also the addition of 50 nM of anti-CD10 TM or anti-CD19 TM. The cell-free supernatants were then collected and the cytokines were analyzed by Enzyme-Linked Immunosorbent Assay (ELISA). Data is shown as mean ± SD of three independent T-cell donors (** p < 0.0021, *** p < 0.0002, **** p < 0.0001; comparison to sample w/o TM; One-way ANOVA with Dunnett’s multiple comparison test).

As shown in Figure 5, UniCAR T-cells are able to significantly increase the production of IFNγ, TNF and IL-2 upon engaging with leukemic cells via the anti-CD10 TM or the anti-CD19 TM; although a higher cytokine release was induced by the latter. As a proof of specificity, the same conditions were applied on a CD10-negative cell line (Molm-13 Luc) in which no significant increase of cytokines was observed. In addition, no, or only marginal, cytokine levels were detected in the control settings in which UniCAR T-cells were co-cultured together with targets cells without TMs, or in combination with the TM but in the absence of target cells.

In addition, we have analyzed a panel of cytokines, including GM-CSF, IFN-α, IFNγ, IL-2, IL-4, IL-5, IL-6, IL-9, IL-10, IL-12, IL-17A and TNF-α, using bead-based technology (MACSPlex assay). We could detect GM-CSF, IFNγ, TNF-α and IL-2 in a comparable level to conventional Enzyme-Linked Immunosorbent Assay (ELISA). However, all other cytokines were undetectable or secreted at very low levels. A selection of cytokines is shown in Supplementary Figure S1.

### 2.6. Killing of Patient-Derived ALL Blasts with Armed UniCAR T-Cells

**Figure 6:**
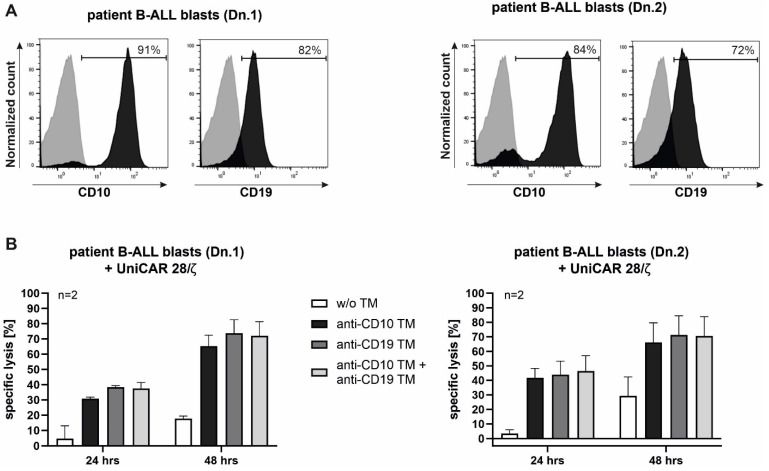
Targeting patient-derived B-ALL blasts with armed UniCAR T-cells. (**A**) Expression of CD10 and CD19 on B-cell acute lymphoblastic leukemia (B-ALL) blasts was detected by flow cytometry after staining with VioBlue-conjugated anti-CD10 or anti-CD19 mAb (dark gray). As a negative control, cells were stained with isotype control (light gray). (**B**) B-ALL blasts were incubated with UniCAR T-cells in the presence of 50 nM anti-CD10 TM, anti-CD19 TM or combination of both TMs at an E:T ratio of 1:1. The viability of ALL blasts was measured using flow cytometry after 24 and 48 h. Results are shown as mean ± SD of two independent T-cell donors for each B-ALL donor (Dn.).

Envisioning clinical translation, we aimed to create more resemblance to natural disease conditions. Therefore, we tested our armed UniCAR T-cells with patient-derived B-ALL blasts, which are characterized by expression of CD10 and CD19 antigens (Figure 6A).

The cytotoxic effect was measured after 24 and 48 h of co-incubation of UniCAR T-cells and the blasts, in the absence or presence of anti-CD10/CD19 TMs, using a flow cytometry-based assay. Due to the modular feature of the UniCAR system, a dual targeting strategy can be applied, where both antigens (CD10 and CD19) can be targeted at the same time by simply applying a combination of both anti-CD10 and anti-CD19 TMs.

As shown in Figure 6B, UniCAR T-cells armed with anti-CD10 TM led to an increase in the specific lysis of the blasts at 24 h with a further increase at 48 h. Some background lysis could be observed in the absence of TM, which could be attributed to the allogenicity between UniCAR T-cells and B-ALL blasts. Both anti-CD10 and anti-CD19 TMs showed comparable potencies. Moreover, combining both TMs did not show a further increase of blasts lysis. In summary, both antigens CD10 and CD19 could be targeted using the same UniCAR T-cells, leading to an increase in the specific lysis of patient-derived leukemic blasts.

## 3. Discussion

The emergence of immune-based therapeutic strategies has started revolutionizing the treatment of cancer. While targeting of solid tumors still faces several hurdles [53,54,55], CAR T-cell targeting CD19 in leukemia and lymphoma has found its way into clinical application, due to high success rates achieved with the therapy [56,57]. However, the clinical use of CAR T-cells has also revealed several drawbacks that need to be addressed in order to optimize their therapeutic use. CD19-specific CAR T-cells can lead to depletion of healthy B-cells and consequently, acquired hypogammaglobulinemia as a result of the on-target/off-tumor effect and the persistent activity of CAR T-cells [58]. In addition, life-threatening toxicities could occur and jeopardize the health of patients [58]. Therefore, there has been a need for improved safety approaches that can provide controllability of the engineered T-cells. Indeed, this has been shown with our switchable UniCAR system, not only in pre-clinical settings but also in a recent phase-1 clinical study [45,49].

Some of the ALL patients treated with anti-CD19 CAR T-cells had partial responses, resistance or even relapse [59]. Targeting single antigens (e.g., CD19) can be associated with the emergence of either altered forms of the target antigen or completely antigen-negative leukemic clones, leading to ineffectiveness of the treatment [4,60]. Therefore, other targets, such as CD20 or CD22, are being investigated to allow subsequent or co-targeting of leukemic cells [61,62,63,64]. In this study, we investigated the targeting of CD10, a common ALL antigen, which is expressed in several types of leukemia and lymphoma. Despite the wide use of CD10 as a diagnostic and prognostic marker in B-cells and other malignancies [14,19,29,37,65], its potential as a target for immunotherapy is understudied.

We have previously described in detail the successful targeting of CD19 on B-ALL cell lines using switchable UniCAR technology [44]. In this study, we describe the design and characterization of a novel anti-CD10 TM, which is based on an anti-CD10 mAb. Although the novel TM is monovalent, it was shown to keep its binding ability to CD10-expressing cells. We have also demonstrated that the TM-armed UniCAR T-cells can induce specific and significant lysis of B-ALL cell lines. In fact, a nanomolar concentration of the TM was sufficient to activate the UniCAR T-cells to exert a cytotoxic effect and to secrete pro-inflammatory cytokines.

We also compared the anti-CD10 TM with our previously-established anti-CD19 TM. As observed, the maximum cytotoxic effect of UniCAR T-cells, armed with anti-CD10 TM, was comparable to that of anti-CD19 TM, but with a higher secretion of pro-inflammatory cytokines induced by the latter. Combining both TMs simultaneously did not appear to further enhance the maximum lysis of primary ALL blasts under the applied conditions. This was actually expected, since the majority of blasts expressed CD10 and CD19 and could already be effectively eliminated upon monospecific targeting. However, we have shown that the same UniCAR T-cells can be efficiently redirected with either anti-CD10 and/or anti-CD19 TMs, which might be helpful to overcome tumor escape variants and tumor heterogeneity, enabling an overall enhanced anti-tumor effect in patients.

In summary, efficient elimination of B-ALL cells can be achieved by targeting CD10 using UniCAR technology. Importantly, the modular nature of the UniCAR system provides the ability to switch the UniCAR T-cells on and off, and thereby it allows the sparing of healthy B-cells after termination of the treatment, and also aids in reducing the risk of toxicities, while granting the flexibility to target several antigens on B-ALL cells.

## 4. Materials and Methods

### 4.1. Cell Culture

Nalm-6 cells and Molm-13 were purchased from DSMZ (Braunschweig, Germany), and cultured in RMPI medium containing 10% FCS in addition to 100 μg/mL penicillin/streptomycin, 1 mM sodium pyruvate, 1% non-essential amino acids and 2 mM N-acetyl-L-alanyl-L-glutamine (Sigma Aldrich, Darmstadt, Germany). The cells were used without further authentication. Nalm-6 cells and Molm-13 cells were transduced to express the firefly luciferase (*Photinus pyralis*) using a lentiviral vector, resulting in Nalm-6 Luc and Molm-13 Luc, as described previously [50]. TM-producing 3T3 cell lines were cultured in DMEM medium, containing 10% FCS, 100 μg/mL penicillin/streptomycin and 1% non-essential amino acids (Sigma Aldrich). Cells were kept at 37 °C with 5% CO_2_ and passaged twice per week.

Patient-derived frozen bone marrow mononuclear cells (BMNCs) were quickly thawed and then incubated for 1 h at 37 °C in RPMI 1640 medium, containing 5% FCS, 2500 U/mL Heparin (Biochrom GmbH) and 2000 U/mL DNAse (Sigma Aldrich). Afterwards, the cells were cultured in StemSpan^TM^ medium (STEMCELL Technologies GmbH, Cologne, Germany), containing 2% FCS, 100 μg/mL penicillin/streptomycin, 2 mM N-acetyl-L-alanyl-L-glutamine, and supplemented with 10 ng/mL of fms-related tyrosine kinase 3 ligand (FLT3-L), 10 ng/mL stem cell factor (SCF), 10 ng/mL thrombopoietin (TPO) and 10 ng/mL interleukin-3 (IL-3).

### 4.2. Expression and Purification of Recombinant Anti-CD10 Target Modules

For the construction of CD10 TMs, the V_H_ and V_L_ domains sequences of anti-CD10 mAb clone MEM-78 were used. To determine the sequences of the variable domains, cDNA of MEM-78 hybridoma cells was amplified with Advantage_HF2 PCR Kit (Clontech Laboratories, Inc., Mountain View, CA, USA), with the forward primer (5′-TTTTTGGATCCSARGTNMAGCTGSAGSAGTCWGG-3′) and the reverse primer (5′-GGAAGATCTATAGACAGATGGGGGTGTCGT-3′) for the detection of the V_H_ domain, and the primers set: (Forward: 5′-TGGAYTYCAGCCTCCAGA-3′) and (Reverse: 5′-CGACTAGTCGACTGGTGGGAAGATGGATACAG-3′) for the V_L_ domain. The amplified sequences were then cloned into the plasmid pGEM^®^-T Easy, as described by the manufacturer (Promega GmbH, Mannheim, Germany). Afterwards, the inserts were sequenced (Microsynth Seqlab GmbH, Goettingen, Germany).

The anti-CD10 TM was designed by joining the V_H_ and V_L_ domains via glycine-serine peptide linkers. In addition, we fused the E5B9 and the 6xHis tag at the C-terminus. This in-silico designed nucleotide sequence was then synthesized by Eurofins Genomics GmbH (Ebersberg, Germany). The synthesized sequence was inserted into the lentiviral vector p6NST50 via the restriction enzymes NheI/MssI (insert digestion) and XbaI/HpaI (vector digestion) (ThermoFisher Scientific, Hennigsdorf, Germany). The anti-CD10 TM was expressed in 3T3 cells, which were generated by transduction with lentiviral particles encoding the TM sequence. Afterwards, the supernatants of transduced 3T3 cells were collected, and the TMs were purified with Ni-NTA affinity columns (Qiagen, Hilden, Germany) through the C-terminal 6xHis tag, followed by analysis with SDS-PAGE, staining with Quick Coomassie^®^ Stain (SERVA Electrophoresis GmbH, Heidelberg, Germany) or immunoblotting, as described previously [49,50].

### 4.3. Isolation and Genetic Modification of T-Cells

T-cells were isolated from buffy coats that were obtained from the German Red Cross (Dresden, Germany) with the informed consent of voluntary healthy donors. The peripheral blood mononuclear cells (PBMCs) were isolated via density gradient centrifugation. Subsequently, the T-cells were magnetically labeled and separated using the Pan T Cell Isolation Kit and autoMACS^®^ Pro Separator (Miltenyi Biotec GmbH, Bergisch Gladbach, Germany). The detailed design and generation of the genetically modified UniCAR T-cells and the control T-cells (Vector and UniCAR stop control) were described in detail previously [49,51,52]. During transduction and expansion, T-cells were cultured with IL-15, IL-7 (ImmunoTools, Friesoythe, Germany) and Proleukin^®^ (Novartis Pharmaceuticals, Basel, Switzerland). However, around 24 h before the assays, genetically modified T-cells were cultured in RPMI complete medium without the cytokines.

### 4.4. Cytokine-Release Assay

The detection of cytokines IFNγ, TNF, IL-2 was performed on cell-free supernatants from a co-culture of 1 × 10^4^ Nalm-6 cells with 5 × 10^4^ UniCAR T-cells in the presence, or absence, of 50 nM of either anti-CD10 or anti-CD19 TM in a total volume of 200 µL medium. The cytokine detection was performed using ELISA (BD Biosciences, Heidelberg, Germany), according to the manufacturer’s instructions. A standard curve, ranging from 7.8 to 500 pg/mL, was used in order to quantify each cytokine. Samples were diluted to make sure they fall below the maximum of the standard curve.

Alternatively, a panel of cytokines were measured in the supernatants using MACSPlex cytokine 12 kit (Miltenyi Biotec GmbH), according to the manufacturer’s instructions, and were analyzed using a MACSQuant^®^ Analyzer and MACSQuantify^®^ software (Miltenyi Biotec GmbH). A standard curve, ranging from 3.2 to 10,000 pg/mL, was used in order to quantify the cytokines.

### 4.5. Flow Cytometry Analysis

In order to detect binding of the anti-CD10 TM to CD10 extracellular domain, 1 × 10^5^ Nalm-6 Luc or Molm-13 Luc cells were first incubated with 5 µL of FcR blocking Reagent (Miltenyi Biotec GmbH), and then stained with anti-CD10 TM (2 µg/50 µL PBS/2% FCS) for 1 h. Thereafter, the cells were washed with PBS and incubated with the anti-La mAb (clone 5B9), directed against the E5B9 epitope (50 µL of 5 µg/mL, 1 h). This was followed by washing with PBS and 30 min incubation with Alexa Fluor 647-labeled goat anti-mouse IgG Ab (Life Technologies, ThermoFisher Scientific). Binding of anti-CD19 TM was performed as described above. For detection of CD10 and CD19 on the cell lines or on the patient-derived B-ALL blasts, 1 × 10^5^ cells were stained with VioBlue-conjugated anti-human CD10 or CD19 REAfinity^TM^ mAbs or isotype control (REA Control Antibody (S), human IgG1, VioBlue^®^, REAfinity^TM^, 1 µL Ab/30 µL PBS/2% FCS, 30 min) (Miltenyi Biotec GmbH). Finally, samples were analyzed using a MACSQuant^®^ Analyzer, MACSQuantify^®^ software (Miltenyi Biotec GmbH) and FlowJo^TM^ Software (FlowJo, Ashland, OR, USA). All stainings were confirmed at least twice.

### 4.6. Cytotoxicity Assays

For luminescence-based cytotoxicity assay, cancer cells expressing the firefly luciferase were used. UniCAR T-cells were co-cultured for 7 h with Molm-13 Luc or Nalm-6 Luc, at different E:T ratios in the absence, or presence, of 50 nM, or a range of concentrations of the TM as indicated for each experiment. The protocol for determination and calculation of specific lysis of tumor cells was described previously in detail [47].

A flow cytometry-based cytotoxicity assay was used for the detection of the viability of patient-derived B-ALL blasts. Therefore, the blasts were labeled with eBioscience^TM^ cell proliferation dye eFluor^TM^ 670 (Thermofisher Scientific). After 24 and 48 h of co-culture with UniCAR T-cells and 50 nM of the TMs, the viable eFluor-labeled blasts were identified by adding 1 μg/mL of propidium iodide/PBS solution (ThermoFisher Scientific), and analyzed with MACSQuant^®^ Analyzer and MACSQuantify^®^ software (Miltenyi Biotec GmbH). The percentage of living cells was calculated using the following equation: (cell count/mL (sample)/cell count/mL (maximum)) ×100%, where the maximum is the cell count in the wells containing only leukemic cells without the addition of UniCAR T-cells or TM. Then, the percentage of lysis was calculated as follow: 100%-living cells%.

### 4.7. Statistical Analysis

Statistical analysis was done using Microsoft World Excel 2017 and GraphPad Prism 9.0 (La Jolla, CA, USA). Statistical significance was determined by one-way ANOVA with Dunnett’s multiple comparison test, or two-way ANOVA with Bonferroni’s multiple comparison. P-values below 0.033 were considered statistically significant.

## Data Availability

The data presented in this study are available upon request from the corresponding authors.

## Supplementary Materials

The following supporting information can be downloaded at: www.mdpi.com/article/10.3390/ijms23094920/s1.
