# Supplementary material for: Targeting CD10 on B-Cell Leukemia Using the Universal CAR T-Cell Platform (UniCAR)
Source: Int J Mol Sci. 2022 Apr 28;23(9):4920. doi: 10.3390/ijms23094920 (PMC9105388; doi:10.3390/ijms23094920)
Supplement: Supplementary file 1 [file ijms-23-04920-s001.zip › ijms-1695281-supplementary.pdf]

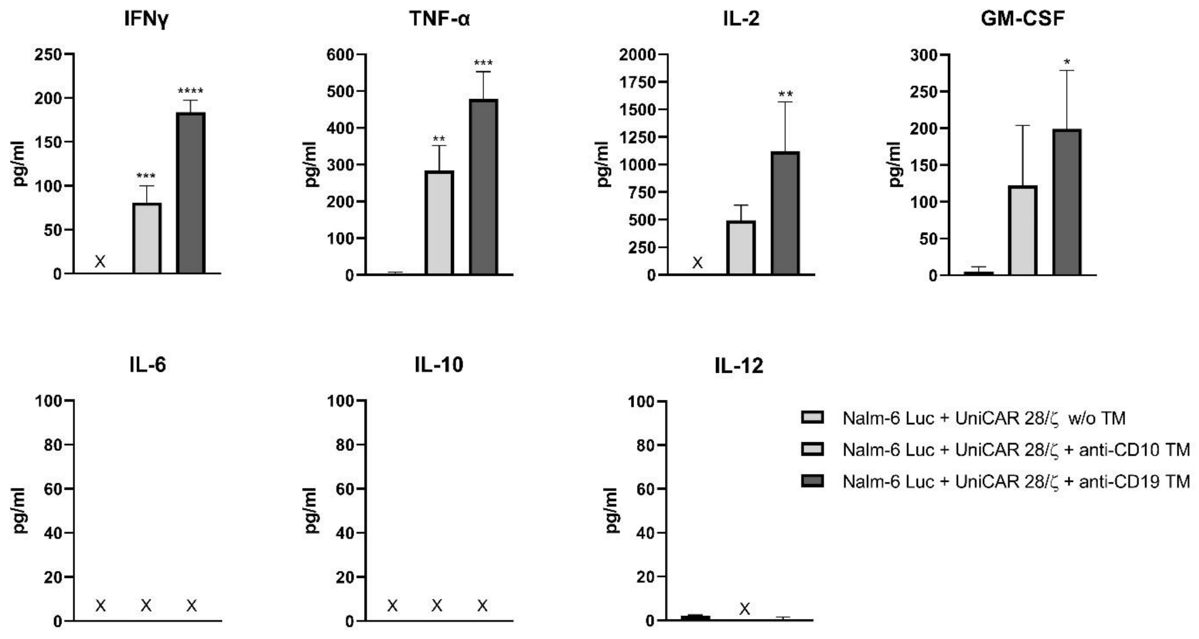

Figure S1. Cytokine release by UniCAR T-cells armed with anti-CD10 TM analyzed using MACSPlex technology. UniCAR T-cells were co-cultured for 7 hrs with Nalm-6 Luc cells at 5:1 E:T ratio in the absence or presence of 50 nM of anti-CD10 TM or anti-CD19 TM. The cell-free supernatants were then collected and the cytokines were analyzed by MACSPlex cytokine kit. Data is shown as mean ± SD of three independent donors (*$p < 0.0332$, **$p < 0.0021$, ***$p < 0.0002$, ****$p < 0.0001$; comparison to sample w/o TM; One-way ANOVA with Dunnett's multiple comparison test).
